# A novel *POLH* mutation causes XP-V disease and XP-V tumor proneness may involve imbalance of numerous DNA polymerases

**DOI:** 10.3892/ol.2013.1604

**Published:** 2013-10-07

**Authors:** JIA GUO, GUILAN ZHOU, WENFENG ZHANG, YALING SONG, ZHUAN BIAN

**Affiliations:** 1 State Key Laboratory Breeding Base of Basic Science of Stomatology and Key Laboratory of Oral Biomedicine Ministry of Education, School and Hospital of Stomatology, Wuhan University, Wuhan, Hubei 430079, P.R. China; 2 Department of Endocrinology, Renmin Hospital of Wuhan University, Wuhan, Hebei 430060, P.R. China

**Keywords:** *POLH*, mutation, Xeroderma pigmentosum, XP-V

## Abstract

Xeroderma pigmentosum variant (XP-V) is a subtype of xeroderma pigmentosum (XP) disease with typical pigmentation and types of cancer in the oral maxillofacial and other sun-exposed regions. Few factors of tumor proneness in XP-V have been completely elucidated with the exception of the *POLH* [which encodes DNA polymerase η (pol η)] mutation. The aim of the present study was to identify the *POLH* mutation in an XP-V patient and to explore the roles of specific additional polymerases in XP-V tumor proneness. The *POLH* gene was sequenced in the patient and the expression of pol η, ι, κ, θ and ζ was tested in XP-V tumor cells and cell lines, as well as in HeLa cells with *POLH* knockdown. The results revealed a novel, large homozygous deletion of *POLH* (del exon 5–9) in the patient. Lower expression of pol κ, θ and ζ were observed in the XP-V cells and similar changes were observed in HeLa cells with *POLH* knockdown. Consistent with XP-V tumor cells, following UV irradiation, the expression of pol κ and θ presented was significantly increased in the XP-V cell lines compared with that in the normal control cells. The unusual expression of other polymerases, besides pol η, identified in the present study indicated that these polymerases may also be key in XP-V cells genetic instability, which accelerates tumor formation.

## Introduction

Xeroderma pigmentosum (XP) is a rare autosomal recessive disorder characterized by extreme sensitivity to sunlight, resulting in sunburn, pigmentation and cancer of exposed skin, particularly in the oral maxillofacial region (1). XP has eight subtypes based on the gene that is affected, namely XP-A, XP-B, XP-C, XP-D, XP-E, XP-F, XP-G and Xeroderma pigmentosum variant (XP-V) (2). XP-V is particularly common, with an incidence rate of 21% (3). Clinical features of XP-V (MIM 278750) include sensitivity to sunlight and sunlight-induced lesions. Significant pigmentation, dryness and roughness commonly occur in sun-exposed skin. Photophobia and corneal opacification are often present in patients (2). The greatest danger for the survival of XP patients is the recurrence of cancer (1). Pigmentation in exposed areas accelerates the malignancy of melanocytes and keratinocytes and eventually leads to tumors, such as multiple basal and invasive squamous cell carcinomas and melanomas (3).

Since mutations in the *POLH* gene (MIM 603968) were first reported in XP-V cell lines in 1999, a number of mutations in the *POLH* gene have been identified in XP-V patients from various populations (4–13). The human *POLH* gene encodes polymerase η (pol η), a homolog of the yeast Rad30 protein, which belongs to the polymerase family Y and is key in translesion DNA synthesis (TLS) (14,15). Although XP-V patients often suffer from a number of various types of skin cancer and a single case usually has more than one category of tumor (8), little is known concerning factors affecting tumor proneness in XP-V besides the *POLH* mutation. Previous studies have revealed that the expression of numerous oncogenes are altered in XP skin tumors (16), which has indicated that XP-V tumor development may involve the unusual expression of numerous genes as well (2). However, to date, no previous studies have explored the expression patterns of other genes besides *POLH* and *POLI* in XP-V tumors (6). XP-V skin cancer proneness is mainly due to genetic instability caused by defective TLS (17), and polymerases, such as pol ζ, κ and ι, are responsible for cyclobutane pyrimidine dimer (CPD) TLS (18). An additional polymerase, pol θ, has the same function as pol η, which is to generate A/T mutations during somatic hypermutation of Ig genes (19). As all these polymerases have been confirmed to bypass DNA damage, with functions similar to those of pol η, it may be deduced that the genes *REV3L*, *POLK*, *POLI* and *POLQ,* encoding pol ζ, κ, ι and θ, respectively, may also have altered expression and contribute to mutagenesis in XP-V tumors.

The present study reports a novel *POLH* mutation and its associated phenotypic features in an XP-V patient, as well as the expression of genes encoding pol η, ζ, κ, ι and θ in XP-V lip tumor cells, XP-V cell lines and HeLa cells in which *POLH* has been knocked down. The observations indicate a possible correlation between several TLS polymerases and genetic instability in XP-V tumor proneness.

## Materials and methods

### Enrollment of subjects

The study protocol was approved by the institutional review board at the School of Stomatology, Wuhan University (Wuhan, China) and informed written consent was obtained from all participants or their guardians. A 65-year-old male was diagnosed with XP disease at the Hospital of Stomatology, Wuhan University. XP-V tumor cells (epithelial cells) were obtained from the lip tumor (squamous cell carcinoma) of the patient, and control epithelial cells were obtained from the normal lip tissue of three children undergoing cleft lip repair surgery at the Hospital of Stomatology, Wuhan University. Blood samples (1–3 ml) were obtained from the diagnosed patient and 96 normal individuals from the local population.

### Polymerase chain reaction (PCR) and DNA sequencing

Genomic DNA was isolated by the standard SDS proteinase K salt chloroform technique (20). Based on a candidate gene approach, all exons, candidate introns and the majority of flanking sequences (GenBank *POLH* accession, NG_009252) were amplified by PCR. Primers used for PCR were made by Sangon Biotech Co., Ltd. (Shanghai, China). The sequence of primers are listed in Table I and the distribution of primers for candidate introns are shown in Fig. 2C. PCR products were analyzed by 2% agarose gel electrophoresis in 0.5X TBE buffer (Bioprimacy Co., Ltd., Wuhan, China) at 5 V/cm and were sequenced by an ABI 3730xl DNA analyzer (Applied Biosystems, Carlsbad, CA, USA).

### Cell culture and cDNA extraction

All cells, including XP-V tumor cells, normal cells, XP-V fibroblast cell lines, human skin fibroblast (HSF) cell lines and HeLa cells, were cultured in DMEM supplemented with 20% FBS (HyClone, Logan, UT, USA). HeLa and HSF cells were purchased from the cell bank of the Chinese Academy Of Sciences (Beijing, China). XP-V fibroblast cell lines (XP30RO and XP1SF) were purchased from Coriell Institute (Camden, NJ, USA). The cells were incubated at 37°C in 5% CO_2_ and the total RNA kit (Omega Bio-Tek, Norcross, GA, USA) was used to extract RNA from each sample. The RNA was used to synthesize cDNA using the First Strand cDNA Synthesis kit (Thermo Fisher Scientific, Waltham, MA, USA).

### Vector construction, transfection and fluorescence detection for subcellular localization

The following primers were purchased from Sangon Biotech Co., Ltd., and were used to amplify the *POLH* coding cDNA (GenBank POLH accession, NM_006502) from XP-V tumor cells and control cells: Forward (F): 5′-ATGCTCGAGCAATGGCTACTGGAC AGGATCG-3′ and reverse (R): 5′-ACGGAATTCCCTGAG GGCAGCACTAATGT-3′. The mutant and wild-type *POLH* cDNA were separately inserted into pEYFP-C1 vectors (Clontech Laboratories, Inc., Mountain View, CA, USA). The vectors were separately transfected into HeLa cells using Lipofectamine 2000 (Invitrogen Life Technologies, Carlsbad, CA, USA) to test whether the mutant pol η had altered subcellular localization. Visualization of YFP-tagged pol η was performed under a fluorescence microscope (DM4000D; Leica, Wetzlar, Germany) at 48 h following transfection. Cells were stained with Hoechst 33258 to mark the nuclei (Sigma-Aldrich, St. Louis, MO USA).

### Knockdown of POLH expression in HeLa cells

Expression of DNA pol η in HeLa cells was knocked down by transfection with polymerase-specific siRNA with the following sequence: 5′-GCCCUUCUUUAAGCAGAAATT-3′. siRNA were purchased from GenePharma Co., Ltd. (Shanghai, China).

### Real-time PCR (qPCR)

*POLH*, *POLI*, *POLK*, *POLQ* and *REV3L* genes encoding the DNA polymerases pol η, ι, κ, θ and ζ, respectively, were examined. Specifically, the expression levels of these genes were compared between XP-V tumor cells and normal control cells. In addition, changes in the expression of these genes upon UV irradiation were also assayed. *GAPDH* was used as an internal control for the normalization of RNA levels. HeLa cells, with and without *POLH* knockdown, were used to verify the changes in expression. For UV irradiation, cells were exposed to UV-C light (5 J/m^2^) and were continued to be cultured normally for 24 h thereafter. The dose of irradiation was selected according to its effect on cells, which has evident toxicity but maintains an appropriate level of cell activity (8,18). The mRNA of HeLa cells with *POLH* knockdown were isolated 24 h following transfection with siRNA. Cells that received UV irradiation were irradiated at 24 h following siRNA transfection, when the expression of *POLH* had evidently been knocked down. Cell mRNA was isolated at 24 h following UV irradiation. Each sample was tested in triplicate. All data are expressed as the mean ± standard error. qPCR was performed using the SYBR^®^ Premix Ex Taq™ II reagent kit (Takara Bio, Inc., Shiga, Japan). The ABI 7500 Real-time PCR System (Applied Biosystems) was used to analyze the data by the ΔΔCt method as described previously (21). To identify the differences in gene expression, samples of tumor and HeLa cells without *POLH* knockdown were defined as the reference samples, and the mRNA quantity of all tested genes in the reference sample was defined as ‘1.0’. Student’s t-test was used to compare the relative expression levels between tumor and normal cells and between HeLa cells with and without *POLH* knockdown. Statistical analyses were performed by SPSS (SPSS Inc., Chicago, IL, USA). P<0.05 was considered to indicate a statistically significant difference. The following primers were used to analyze the mRNA levels of the five genes studied: F: 5′-AGTTCGTGAGTCCCGTGGG-3′ and R: 5′-GCTTGGCAA CAAGTCTGCC-3′ for *POLH*; F: 5′-GTCGTGAGAGTCGT CAGTGC-3′ and R: 5′-GCT TGCCAGAGCGTGAAGTA-3′ for *POLI*; F: 5′-AGCCATGCCAGGATTTATTG-3′ and R: 5′-GGATCGTTCATGCTCACTCA-3′ for *POLK*; F: 5′-AAAGAACTCCTGGAAGTGATGGA-3′ and R: 5′-GCCAAGACCCGAATGAGACC-3′ for *POLQ*; F: 5′-CCGTGTCCGTGGAAATCTCC-3′ and R: 5′-GTGGGGCTCTCATCTGGGAT-3′ for *REV3L*; and F: 5′-TCATGGGTGTGAACCATGAGAA-3′ and R: 5′-GGCATG GACTGTGGTCATGAG-3′ for *GAPDH*.

### Western blot analysis

All cells were lysed with cell lysis buffer (Beyotime, Shanghai, China) supplemented with 1 mM PMSF (Bioprimacy Co., Ltd.). Protein extracts were separated by 10% SDS polyacrylamide gel (Beyotime) electrophoresis and then electrophoretically transferred to polyvinylidene fluoride membranes (Bio-Rad Laboratories, Inc., Hercules, CA, USA). Following blocking with 5% skimmed milk, the membranes were separately incubated with rabbit polyclonal antibodies against human pol η (1:2,000), ι (1:8,000) and θ (1:1,000) (all Abcam, Cambridge, UK), and mouse monoclonal antibodies against human pol κ and ζ (both 1:1,000; Abcam) and GAPDH (1:1,000; Santa Cruz Biotechnology, Inc., Santa Cruz, CA, USA) at 4°C overnight. Secondary anti-mouse or anti-rabbit IgGs conjugated to horseradish peroxidase (Santa Cruz Biotechnology, Inc.) were incubated with the membranes for 1 h at room temperature at 1:8,000 dilution in PBS containing 0.1% Tween 20 (Bioprimacy Co., Ltd.). The blots were developed using SuperSignal West Pico chemiluminescent substrate (Pierce Biotechnology, Inc., Rockford, IL, USA).

### Viability of UV-irradiated cells

Viability of UV-irradiated cells was determined by conducting the CellTiter-Glo luminescent cell viability assay (Promega Corporation, Madison, WI, USA), which measures the metabolic activity of cells by detecting cellular ATP (18).

## Results

### Patient characteristics

**Figure 1:**
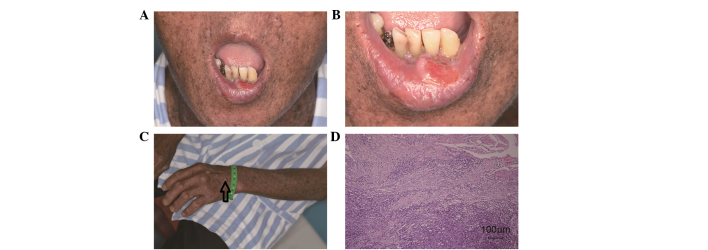
Clinical phenotypes of the XP-V patient. (A) Marked pigmentation was found to be widely distributed over the maxillofacial region. (B) Tumor was visible on the lower lip with an ulcer on the surface. (C) Freckling and recurrent ulcers, indicated by the black arrow, were identified on the dry and rough skin of the limbs and hands. (D) Histological examination with hematoxylin and eosin staining showed squamous cell carcinoma under the microscope (magnification, ×10). XP-V, xeroderma pigmentosum variant.

The typical signs of the sun damage phenotype were present in the investigated patient. The patient, a farmer who had previously had prolonged exposure to sunlight without careful protection, separately underwent lingual and temporal tumor resection 20 years ago. Freckling was widely distributed on the exposed skin and recurrent skin ulcers were identified on the patient’s hands and limbs (Fig. 1C). Squamous cell carcinoma was diagnosed on the patient’s lower lip (Fig. 1A, B and D). In addition, the right eye had suffered from conjunctivitis for a number of years, which developed into glaucoma six years ago. No neurological abnormalities were identified in the patient. The patient has no siblings and the parents of the patient are consanguineous and normal. Other members of the patient’s family do not share any of these phenotypes.

### PCR and sequencing of the cDNA

**Figure 2:**
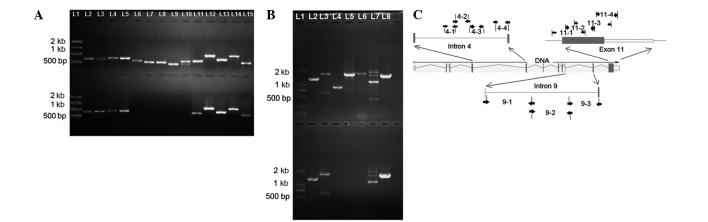
PCR results analyzed by gel electrophoresis and the distribution of primers for long exons and candidate introns. (A) Results of PCR amplification of *POLH* exons analyzed by agarose gel electrophoresis showed that the patient had no PCR products between exons 5 and 9. The upper lanes represent the normal controls while the lower lanes represent the XP-V patient. Lanes 1, DNA maker; 2–11, PCR products of exon 1–10; and 12–15, four PCR products of four pairs of primers for exon 11. (B) PCR products of introns 4 and 9 analyzed by agarose gel electrophoresis. The upper lanes represent normal controls and the lower lanes represent the XP-V patient. Lanes 1, DNA maker; 2–5, PCR products of primers 4-1 to 4-4 in intron 4; and 6–8, PCR products between primers 9-1 and 9-3 in intron 9. No PCR products were formed for primers 4-3, 4-4 and 9-1 in the patient’s sample. (C) Distribution of primers in introns 4 and 9 and exon 11. Gray blocks indicate coding exons and white blocks indicate UTR regions, while gray lines indicate introns. Each thick arrow represents a primer and the numbers between the arrows correspond with the number of primers in Table I. The sites of arrows on the DNA strand indicate the approximate sites of the primer binding on the DNA sequence. PCR, polymerase chain reaction; XP-V, xeroderma pigmentosum variant.

**Figure 3:**
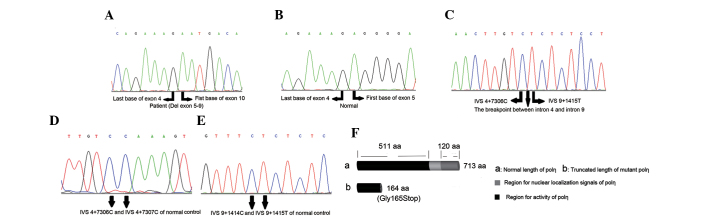
*POLH* sequencing results. (A) Sequencing *POLH* cDNA from the XP-V patient verified the large deletion between exons 5 and 9. (B) Normal *POLH* cDNA sequence at the boundary of exons 4 and 5. (C) Breakpoints of the deletion mutation in *POLH* genomic DNA obtained from the XP-V patient. (D and E) Breakpoints of introns 4 and 9 in normal controls. (F) Full-length normal pol η and predicted size of the truncated protein. XP-V, xeroderma pigmentosum variant; aa, amino acid; pol η, polymerase η.

The genomic DNA of the patient yielded no bands between exons 5 and 9 of the *POLH* gene, whereas intact bands were observed in the DNA from the normal controls in PCR reactions (Fig. 2A). Based on this observation, it has been deduced that there is a large homozygous deletion with breakpoints in introns 4 and 9 that disrupts the intervening exons. Sequencing of the cDNA obtained from tumor cells verified the existence of such a deletion (Fig. 3A and B). This deletion causes an early termination at amino acid (aa) site 165 in the peptide (Fig. 3F). Primers were designed for introns 4 and 9 to identify the breakpoints of the deletion (Fig. 2C; Table I). Unlike with normal cells, the patient exhibited no bands following the region of primer 4-2 in intron 4 and was defective within the region of primer 9-1 in intron 9 (Fig. 2B). Thus, the breakpoints were narrowed down to the region of primers 4-3 and 9-1. To determine the specific breakpoints, the primers 4-2 F and 9-1 R were used as the upstream and downstream primers, respectively, to amplify the patient’s genomic DNA. A 2.8-kb band was amplified from the DNA of the patient, but not from the DNA of normal controls (data not shown). Sequencing of the 2.8-kb band revealed the exact breakpoint in intron 4 to be 7306 bp from exon 4 and the breakpoint in intron 9 to be 1415 bp from exon 9 (Fig. 3C–E). The same introns of *POLH* were also examined in 96 normal individuals to rule out the possibility of this deletion being a polymorphism (data not shown).

### Expression of the POLH-pEYFP-C1 vector in HeLa cells

**Figure 4:**
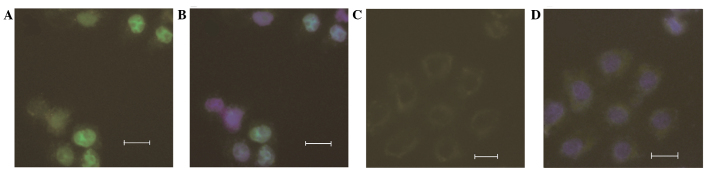
Expression of the *POLH*-pEYFP-C1 vector in HeLa cells. Green fluorescence in the cells indicates the localization of pol η and blue fluorescence indicates the distribution of nuclei in HeLa cells. (A) Normal pol η was localized in the nucleus and cytoplasm. (B) Blue fluorescence indicates the distribution of nuclei in the same field of view as observed in A. (C) Green fluorescence was observed only in the cytoplasm, which indicated that mutant pol η localized in the cytoplasm but not in the nucleus. (D) Blue fluorescence shows the distribution of nuclei in the same field of view as observed in C (scale bar, 20 μm). Pol η, polymerase η.

The mutant and wild-type *POLH*-pEYFP-C1 vectors were transfected into HeLa cells to examine the effect of the deletion found in the patient on the function of *POLH*. Fluorescence detection showed that while wild-type pol η localized in the cytoplasm and nucleus, the truncated pol η, encoded by mutant *POLH,* did not localize in the nucleus. The intensity of fluorescence in the nucleus was higher than that in the cytoplasm of the wild-type *POLH*. In addition, the level of YFP-tagged mutant protein was lower than that of the wild-type protein in those cells (Fig. 4A–D).

### qPCR analyses

**Figure 5:**
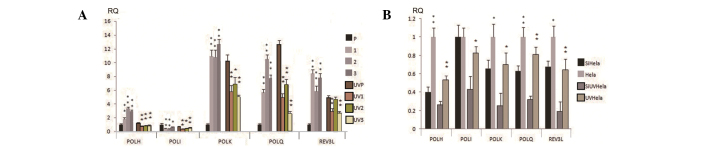
Results of qPCR analyses of *POLH*, *POLI*, *POLK*, *POLQ* and *REV3L* in control and UV-exposed cells. (A) Sample P, XP-V tumor cells from the patient; samples 1–3, normal cells from three controls; and samples UVP, UV1, UV2 and UV3, samples P, 1, 2 and 3 that underwent UV irradiation, respectively. Prior to UV irradiation, lower expression of *POLH*, *POLK*, *POLQ* and *REV3L* and higher expression of *POLI* was observed in XP-V tumor cells (sample P) compared with normal controls (samples 1–3) (P<0.05). Following UV irradiation, expression of *POLK*, *POLQ* and *REV3L* was found to have significantly increased in XP-V tumor cells. All other genes, with the exception of *REV3L,* had higher expression in tumor cells (sample UVP) than in normal cells (samples UV1-UV3) (P<0.05). (B) Tumor samples (sample P) and HeLa cells were set as reference samples and the RNA quantity of each tested gene was defined as 1.0. Prior to UV irradiation, *POLH*, *POLK*, *POLQ* and *REV3L* had lower expression in the SiHeLa sample than in the HeLa sample (P<0.05). Following UV irradiation, all tested genes had lower expression in the SiUVHeLa sample than in the UVHeLa sample (P<0.05). ^*^Statistically significant difference between samples P and 1, 2 and 3, and between SiHeLa and HeLa cells; ^*^statistically significant difference between samples UVP and UV1, UV2 and UV3, and between SiUVHeLa and UVHeLa cells. One symbol indicates P<0.05 and two symbols indicates P<0.01. qPCR, real-time polymerase chain reaction; XP-V, xeroderma pigmentosum variant; RQ, mRNA relative quantity; HeLa, HeLa cells without *POLH* knockdown; SiHeLa, HeLa cells with *POLH* knockdown; SiUVHeLa, UV-irradiated HeLa cells with *POLH* knockdown; UVHeLa; UV-irradiated HeLa cells without *POLH* knockdown.

qPCR results revealed that the mRNA quantities of *POLH*, *POLK*, *POLQ* and *REV3L* in XP-V tumor cells were lower than those of the controls; however, the expression of *POLI* was higher in the XP-V tumor cells in the absence of UV irradiation (P<0.05; Fig. 5A). Upon UV irradiation, all these genes were expressed at higher levels in the tumor cells than in the control cells (P<0.05), with the exception of *REV3L*, which exhibited similar expression levels in the XP-V tumor cells and one of the normal controls (sample UV2) (Fig. 5A). Specifically, expression of *POLK*, *POLQ* and *REV3L* increased in the tumor cells while that of *POLH*, *POLK*, *POLQ* and *REV3L* decreased in the normal controls. Transfection of HeLa cells with polymerase-specific siRNA effectively knocked down the mRNA levels of *POLH* (P<0.01), and expression of all but one (*POLI*) of the tested genes was found to decrease in these cells (P<0.05; Fig. 5B). Detailed data are presented in Table II.

### Western blot analyses

**Figure 6:**
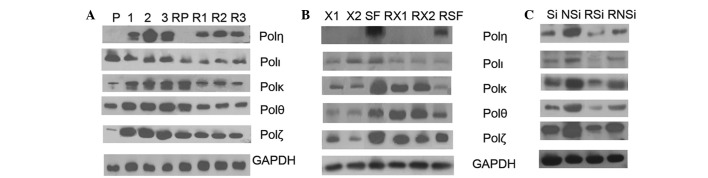
Western blot analysis of pol η, ι, κ, θ and ζ. GAPDH protein levels were used for normalization. (A) Tested polymerases in tumor cells from the XP-V patient and normal cells from three controls, including samples P, tumor cells from the patient; 1–3, normal cells from three controls; and RP, R1, R2 and R3, cells from samples P, 1, 2 and 3 that underwent UV irradiation, respectively. (B) Tested polymerases in XP-V and HSF cell lines, including samples X1 and X2, cells from XP30RO and XP1SF, respectively; SF, cells from HSF cell line; and RX1, RX2 and RSF, cells from samples X1, X2 and SF that underwent UV irradiation, respectively. (C) Tested polymerases in HeLa cells, including samples Si, HeLa cells with *POLH* knockdown; NSi, HeLa cells without *POLH* knockdown; and RSi and RNSi, cells from samples Si and NSi, respectively, that underwent UV irradiation. XP-V, xeroderma pigmentosum variant; pol η, polymerase η.

Concordant with the decrease in the mRNA quantities of pol κ, θ and ζ, their protein levels were also found to significantly decrease in the XP-V tumor cells compared with those of the controls. Following UV irradiation, pol κ and θ were found to be at higher levels in the XP-V tumor cells, whereas pol ι and ζ were at comparable levels in the XP-V tumor cells and normal controls (Fig. 6A). XP-V cell lines (XP1SF and XP30RO) expressing mutant pol η verified these changes when the cell lines were compared with the HSF cell line (Fig. 6B). As the defective pol η lacked the motif normally recognized by its antibody, binding of pol η was not observed in the western blot assays. Similar to the results of the qPCR analyses, HeLa cells subjected to UV irradiation exhibited lower levels of all the tested proteins when *POLH* was knocked down (Fig. 6C).

### Cell viability

The qPCR and western blot analyses revealed that, in the epithelial control, HeLa and HSF cells, expression of all the tested genes was found to decrease following UV irradiation. We hypothesized that this may have been caused by the reduction of cell viability following UV irradiation. Cellular ATP levels were measured to assay the activity of these cells. The viability of the cells was observed to decrease substantially (52±5% in normal epithelial, 47±6% in HSF and 36±4% in HeLa cells) 48 h following UV irradiation, therefore confirming the hypothesis.

## Discussion

Pol η is an essential polymerase that allows cells to bypass a DNA lesion during DNA replication. Although pol η usually shows low fidelity (22) in DNA replication, it replaces conventional polymerases with high fidelity to easily perform TLS in the replication of photodamaged DNA (23). Pol η is the only error-free polymerase reported to bypass the thymine-thymine CPD, a UV-induced DNA damage product (4,23). In XP-V patients with *POLH* mutations, when skin cells have suffered UV irradiation and generate harmful products, the defective pol η does not bypass CPD broken sites and therefore, DNA replication does not continue normally (4,24). As a result, UV lesions are highly mutagenic, leading to skin cancer.

Human *POLH* is located on chromosome 6p21.1 and encodes a peptide of 713 aa. Previous studies have demonstrated that the N-terminal, 511 aa of the polymerase, is necessary for its activity (25) while the C-terminal, 70 aa, is responsible for its nuclear localization and a further 50 aa are required for relocalization following UV irradiation (10). Although a number of missense and small deletion mutations have been found in the *POLH* gene, no previous studies have reported a large deletion spanning greater than one exon in this gene. The present study revealed a large deletion in the *POLH* gene that disrupts the region between exons 5 and 9. This mutation is likely to result in the severe truncation of pol η, with only 164 aa remaining intact and the loss of key regions responsible for activity and nuclear localization (Fig. 3F). Consistent with this hypothesis and previous observations, the mutation was found to lead to alteration in the cellular localization of the protein.

Of note, the XP-V patient investigated in the present study survived longer than the majority of XP patients (26). Although the patient exhibited a severe truncation of the *POLH* gene, the patient did not exhibit more dangerous phenotypes or have a shorter lifespan than other XP-V cases (8). It has been proposed that the phenotype of XP patients depends on their protection against sun toxicity, but not the mutation (2). However, the subject of the present study was a farmer who had been exposed to prolonged sunlight without careful protection, but did not show more severe phenotypes. It has also been reported that XP-V patients often possess relatively mild phenotypes and rarely develop neurological abnormalities compared with other XP patients (2). Therefore, the factors avoiding severe damage in XP-V patients requires further investigation.

Previous studies have demonstrated that *POLI*, *POLK* and *REV3L* are responsible for CPD TLS (18,27,28). Such genetic instability is caused by the combined effect of several unusual polymerases. Knockdown of *REV3L* alone, *POLK* and *POLI* together, or *POLK* and *REV3L* together, has been previously reported to lead to a significant decrease in accurate TLS of XP-V cells (18). In the present study, low expression of *POLH*, *POLK* and *REV3L* was observed in the XP-V tumor cells. This indicates that the low efficiency of TLS contributes to genetic instability in XP-V tumor cells and involves the imbalance of other specific polymerases besides defective pol η. As pol η is the only error-free polymerase in the TLS of CPDs, when it loses its function, other error-prone polymerases may maintain their low expression to avoid mismatch in the replication of CPDs. *POLI*, the only gene with a higher expression in XP-V tumor cells, may compensate for TLS. Consistent with this, low expression of pol κ, ζ and θ was found in the XP-V cell lines and HeLa cells with *POLH* knockdown. As the XP-V tumor often occurs in exposed regions, it is possible that the damaged cells have been exposed to high levels of UV. Considering that the patient in the current study was a farmer with potentially high exposure to irradiation, we hypothesized that this may have been the cause of XP disease. In addition, increased expression of pol κ and ζ was observed in the XP-V cells following strong UV irradiation. When DNA has been damaged by UV irradiation and pol η completely loses its function, pol κ and ζ may compensate for TLS, which may explain the higher expression of these genes in the XP-V tumor cells and cell lines. In the current study, following UV irradiation, all tested genes were found to have a lower expression in the HeLa cells with *POLH* knockdown. This may be due to residual pol η, which may function normally in the TLS of CPDs, leading other genes to maintain a low expression to avoid mismatch in the replication of CPDs.

Based on these observations, we hypothesized that when the XP-V cells suffer strong UV toxicity and generate numerous photodimers, they synthesize more pol κ and ζ to compensate for the defective pol η. However, pol κ and ζ are error-prone, therefore, these polymerases promote mismatch in DNA replication and accelerate the genetic instability (18), which may facilitate tumor formation. If patients avoid UV irradiation, it is likely that the skin cells maintain a low level of pol κ and ζ to prevent error-prone DNA replication. However, low levels of pol η, κ and ζ decrease the efficiency of TLS, and therefore, DNA lesion replication is disrupted as well. It has been previously demonstrated that the upregulation of pol θ perturbs DNA replication, promotes genetic instability and is associated with poor prognosis in breast cancer (29). The significant increase in the expression levels of pol θ observed in the XP-V tumor cells and cell lines following UV irradiation in the present study may also indicate genetic instability.

In summary, the current study reported a novel, large deletion of *POLH* in a XP-V patient. qPCR and western blot analyses of cells expressing mutant *POLH* were conducted. The results indicated that genetic instability in XP-V tumors may arise due to imbalances in DNA polymerases, which may be contributed not only by defects in pol η, but also by the unusual expression of other polymerases. Further investigation is required to clarify the correlation between genotype and resulting phenotype in XP-V, as well as to elucidate the molecular mechanism involved in XP-V tumor formation.

## Figures and Tables

**Table I:** PCR primers of *POLH* genomic DNA.

Exon or intron no.	Size, bp	Forward primer, 5′→3′	Reverse primer, 5′→3′
1	554	GCTGGAGGAGGAGCGTTAGC	ACCTTGGCCCAGTCACTGCT
2	518	TCCATGCTCCCATGCTCATGG	TCCCCATCTCTACCCACCCC
3	574	CTGAACTGCTTTGTTTTGGATTGAA	TGGATGGAGAACATGGGAATTGG
4	568	GTTTGCCTCCGTGATTCTTCCT	TCCTTCCATGTGAGTCCTTGTG
5	448	GCCTATGCTGAAGCTAAGCTGC	TTTCAATGTCCCACTGTCCCT
6	397	CCCACTGGGGATGTTGTGGG	TAAGGACTGGAGCCAGGGGA
7	398	TGTCCTGAACCTTTTGGAGAGCT	TGGGTCACTATGGCCCATCAG
8	343	GGCAGGGGTTTCGTCAGAGG	CCAAAACCTACCCACTGACCCC
9	458	ACCTGATGGCAAACAAGC	CTGGGAGACAGAGTGAGACC
10	471	CCAAACCATTGTCACCCTGG	TTACCCTTTACCTCATTGAAGGACT
11-1	749	GGTTCTCAAGACATAACATCAGCA	AAACAGGGACACACCCTGGA
11-2	543	CAGGGAAGTGGCCCAGCG	TACCGGTACCAGGGAGCCAC
11-3	741	GTCAAAGTACAGGAACTGAGCCCT	GCCTGTGAAGAGATGGGACCG
11-4	417	ACCCCCAGGTTGTTTCTGCC	AGACTCCAAGGCCCACACAC
4-1	1333	AGGGAAGCTTGTGACTTAAGGAAT	CTGAGCATGATTGCTAGCTCTTAT
4-2	1766	ATAAGAGCTAGCAATCATGCTCAG	TTAACTGAACGGGACCACAGA
4-3	910	TATTTCCTGTAGTCCATTTCATGGTA	ATTGTTAAAAGAACATTCTGCAGTCA
4-4	1708	GTTCCTCAGCACAATGGCTTGC	CCCGCAGCTTAGCTTCAGCA
9-1	1813	GTACAATGGTGGCTGTTGCA	GCTACAATACGGCTGAACCTG
9-2	1976	CAGGTTCAGCCGTATTGTAGC	GCAAAGAAACGGGAAGTGCT
9-3	1614	AGCACTTCCCGTTTCTTTGC	CTGAGGCGTTTGTCTCCTTG

**Table II:** Detailed statistics of qPCR results.

	Group A		Group B				Group UVA		Group UVB			
				
Gene	Sample P	SiHeLa	Sample 1	Sample 2	Sample 3	HeLa	Sample UVP	SiUVHeLa	Sample UV1	Sample UV2	Sample UV3	UVHeLa
*POLH*	1±0.12	0.40±0.06	1.81±0.18^a^,^c^	3.34±0.19^a^,^c^	2.90±0.21^a^,^c^	1±0.10^a^,^c^	1.23±0.06	0.26±0.04	0.76±0.09^b^,^c^	0.84±0.08^b^,^c^	0.87±0.09^b^,^c^	0.530±0.05^b^,^c^
*POLI*	1±0.17	1.00±0.13	0.44±0.05^a^,^c^	0.35±0.04^a^,^c^	0.61±0.09^a^,^d^	1±0.10	0.73±0.03	0.43±0.14	0.32±0.07^b^,^c^	0.42±0.10^b^,^d^	0.58±0.03^b^,^c^	0.825±0.07^b^,^d^
*POLK*	1±0.15	0.66±0.09	10.95±0.85^a^,^c^	10.79±1.04^a^,^c^	12.66±0.70^a^,^c^	1±0.14^a^,^d^	10.20±0.91	0.25±0.14	5.75±0.95^b^,^c^	6.88±0.96^b^,^d^	4.98±0.30^b^,^c^	0.700±0.13^b^,^d^
*POLQ*	1±0.15	0.63±0.06	5.67±0.48^a^,^c^	10.52±0.65^a^,^c^	7.71±0.48^a^,^c^	1±0.10^a^,^c^	12.6±0.65	0.32±0.04	4.91±0.57^b^,^c^	6.79±0.83^b^,^c^	2.58±0.33^b^,^c^	0.810±0.08^b^,^c^
*REV3L*	1±0.14	0.68±0.06	8.39±0.58^a^,^c^	5.86±0.75^a^,^c^	7.78±0.63^a^,^c^	1±0.12^a^,^d^	4.97±0.25	0.19±0.10	2.95±0.41^b^,^c^	4.65±0.35	2.73±0.20^b^,^c^	0.640±0.12^b^,^c^

Statistics in Group A, RQ results of sample P (XP-V tumor cells) and SiHeLa cells; group B, RQ results of samples 1, 2 and 3 (normal control cells) and HeLa cells; groups UVA and UVB, RQ results of cells in groups A and B, respectively, following UV irradiation. All data are presented as the mean ± SE.

a Statistically significant difference between samples P and 1, 2, 3, and between SiHeLa and HeLa cells;

b statistically significant difference between samples UVP and UV1, UV2 and UV3, and between SiUVHeLa and UVHeLa cells.

c P<0.01 and

d P<0.05.

qPCR, real-time polymerase chain reaction; RQ, mRNA relative quantity; HeLa, HeLa cells without *POLH* knockdown; SiHeLa, HeLa cells with *POLH* knockdown.
